# Whole-Genome Comparison of Two *Campylobacter jejuni* Isolates of the Same Sequence Type Reveals Multiple Loci of Different Ancestral Lineage

**DOI:** 10.1371/journal.pone.0027121

**Published:** 2011-11-11

**Authors:** Patrick J. Biggs, Paul Fearnhead, Grant Hotter, Vathsala Mohan, Julie Collins-Emerson, Errol Kwan, Thomas E. Besser, Adrian Cookson, Philip E. Carter, Nigel P. French

**Affiliations:** 1 Institute of Veterinary, Animal and Biomedical Sciences, Massey University, Palmerston North, New Zealand; 2 Department of Mathematics and Statistics, Lancaster University, Lancaster, United Kingdom; 3 Animal Health Section, AgResearch, Hopkirk Research Institute, Palmerston North, New Zealand; 4 Department of Veterinary Microbiology and Pathology, Washington State University, Pullman, Washington, United States of America; 5 Rumen Microbiology, Animal Nutrition & Health Capability Group, AgResearch Limited, Grasslands Research Centre, Palmerston North, New Zealand; 6 Institute of Environmental Science and Research Ltd., Wellington, New Zealand; Institut Jacques Monod, France

## Abstract

*Campylobacter jejuni* ST-474 is the most important human enteric pathogen in New Zealand, and yet this genotype is rarely found elsewhere in the world. Insight into the evolution of this organism was gained by a whole genome comparison of two ST-474, *fla*A SVR-14 isolates and other available *C. jejuni* isolates and genomes. The two isolates were collected from different sources, human (H22082) and retail poultry (P110b), at the same time and from the same geographical location. Solexa sequencing of each isolate resulted in 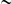
1.659 Mb (H22082) and 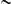
1.656 Mb (P110b) of assembled sequences within 28 (H22082) and 29 (P110b) contigs. We analysed 1502 genes for which we had sequences within both ST-474 isolates and within at least one of 11 *C. jejuni* reference genomes. Although 94.5% of genes were identical between the two ST-474 isolates, we identified 83 genes that differed by at least one nucleotide, including 55 genes with non-synonymous substitutions. These covered 101 kb and contained 672 point differences. We inferred that 22 (3.3%) of these differences were due to mutation and 650 (96.7%) were imported via recombination. Our analysis estimated 38 recombinant breakpoints within these 83 genes, which correspond to recombination events affecting at least 19 loci regions and gives a tract length estimate of 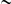
2 kb. This includes a 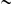
12 kb region displaying non-homologous recombination in one of the ST-474 genomes, with the insertion of two genes, including *ykg*C, a putative oxidoreductase, and a conserved hypothetical protein of unknown function. Furthermore, our analysis indicates that the source of this recombined DNA is more likely to have come from *C. jejuni* strains that are more closely related to ST-474. This suggests that the rates of recombination and mutation are similar in order of magnitude, but that recombination has been much more important for generating divergence between the two ST-474 isolates.

## Introduction

Our understanding of the rate and determinants of bacterial evolution has been revolutionised by the development and application of new tools in molecular biology, genomics and statistical genetics. These include the advent of Next Generation Sequencing [1] and novel approaches to making inference on whole genome data, without the need for time-consuming, full annotation [2]–[4]. These advances have provided new insight into the role of mutation, horizontal gene transfer and selection in the evolution of human and animal pathogens.

*Campylobacter jejuni* and *Campylobacter coli* are major causes of diarrhoeal disease throughout the world [5]. Until recently we had relatively little understanding of the evolution of *Campylobacter spp.* but multilocus sequence typing (MLST) of large collections of isolates, combined with coalescent modelling, has provided new insight into the possible convergence of these two species [6] and revised our estimates of the rate of mutation and recombination [7]. This has led to notable advances in our ability to attribute epidemiologically significant isolate clusters to specific ecological sources, both animal and environmental [8], [9]. However, the seven housekeeping gene MLST scheme, even when supplemented by the addition of one or more hypervariable genes [10]–[12], covers less than 0.5% of the 1.6–1.8 Mb *C. jejuni* genome and therefore provides limited insight into the full extent of genomic variation between seemingly closely-related bacteria.

New Zealand has one of the highest notification rates of human gastroenteritis caused by *C. jejuni* in the world [13]. *C. jejuni* multilocus sequence type 474 (ST-474) is estimated to account for approximately 25–30% of these human campylobacteriosis cases in New Zealand, and is most strongly associated with poultry [9], [14]. The relative rarity of ST-474 in other countries combined with its persistence in both humans and poultry suggests that the population of ST-474 in New Zealand has arisen as a result of clonal expansion from a single common ancestor [15] that evolved from a more ubiquitous, historically-introduced lineage. This is further supported by the presence of multiple Pulsed Field Gel Electrophoresis variants of ST-474 in New Zealand [16]. However, since designation of the ST-474 cluster of related strains is based only upon a sub-region within the seven conserved MLST genes, it is unclear how much variation might exist between any two ST-474 isolates, and how such variation has arisen. To answer these questions we conducted a genome-wide analysis and compared the full gene complement of two ST-474 isolates, one from a retail chicken (P110b), the other from an anonymous human clinical case (H22082) with identical allelic profiles for the seven MLST housekeeping genes and for the hypervariable *fla*A SVR locus [10], [11]. Both were isolated within the same month in the same geographical location in New Zealand. Using Solexa resequencing technology, we describe the differences between the two draft genomes and, by comparing individual genes with those present in other sequenced bacterial genomes, we attempt to identify the most likely ancestral lineage for each gene.

In order to investigate gene lineage, it is necessary to define orthologous genes from a set of genomes. The concepts of the core, accessory, character and pan genomes can be evaluated at the species [17], genus [18], or even at the kingdom level [19]. Estimates in the size of the *C. jejuni* core genome, that is, those genes present in all genomes under investigation, vary depending on the stringency of the parameters used and the samples under investigation, for example ranging from 847 [20] to 1295 [21] in different studies. In contrast, the *Campylobacter* genus core genome has been estimated to comprise 647 genes [18]. In this study we estimate the size of the ‘core genome’ for the thirteen *C. jejuni* isolates considered in this study from orthologous gene clusters. Furthermore, we assess the role of mutation and recombination in determining sequence variation within *C. jejuni*.

## Materials and Methods

### Ethics statement

Ethical approval from the Multi-region Ethics Committee of the Ministry of Health, New Zealand is in place for our work on Campylobacter (application number MEC/10/16/EXP). Ethical approval was not required for the collection of the isolate for clinical diagnostic purposes. Isolates were collected and analysed as part of routine public health investigation activities (as stated below) but approval was sought for subsequent research using these isolates and related surveillance data. Under the 2006 guidelines from the National Ethics Advisory Committee, Ministry of Health, Wellington, New Zealand, “ Ethical Guidelines for Observational Studies: Observational research, audits and related activities”, public health investigations are defined in section 2.4 as investigations that “ explore possible risks to public health, are often of an immediate or urgent nature, and are often required by legislation. For example, investigations into outbreaks or clusters of disease, analyses of vaccine safety and effectiveness, and contact tracing for communicable conditions.” Under these same guidelines, section 3.6 states that “ Public health investigations, as defined above, do not require ethics committee review. This is because they are required for the protection of public health as central parts of public health practice, they are often of an immediate or urgent nature, and they are often required by legislation. Examples of such activities include investigations, undertaken by authorised people, into clusters of disease suspected to be caused by environmental agents and contact tracing.” Section 12.1 also states “ Publication or an intention to publish does not make an activity research rather than an audit or related activity, does not make it a more-than-minimal risk activity, and does not trigger any requirement for ethics committee review.”

### Isolates used in the study

Two isolates of *C. jejuni* were investigated, one isolated from a poultry carcase purchased in Palmerston North, New Zealand on the 28th August 2005 (P110b) and the other from a faecal sample from a human clinical case of campylobacteriosis resident in the same city on the 26th August 2005 (H22082).

### Genomic DNA preparation and sequencing

The Illumina Genome Analyzer was used to sequence the two genomes according to the manufacturer's instructions with read lengths of 36 bp. Briefly, genomic DNA for each of P110b and H22082 was prepared from bacteria grown on blood agar medium (Fort Richard, Auckland, NZ) using a Wizard® Genomic DNA Purification Kit (Promega) according to the manufacturer's instructions. A library for unidirectional sequencing was prepared from 5 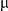
g genomic DNA for each isolate using a single end DNA Sample Prep Kit (Part Number FC-102-1001, Illumina Inc). The genomic DNA was fragmented by nebulisation for 6 minutes at a pressure of 32 psi, end repaired, tailed, adaptor-ligated, fractionated, purified and enriched according to the manufacturer's instructions. A flow cell was prepared for each isolate using the Single Read Cluster Generation Kit (Part Number GD-203-1001, Illumina Inc) and a Cluster Station. Sequencing reactions using 4 pmoles of the library were performed on an Illumina Genome Analyser instrument with a single read 36 cycle SBS sequencing kit (Illumina Inc) to give approximately 3 to 5 million clusters per lane.

### MLST and reference genome sequence data

Information on MLST alleles for *C. jejuni* and *C. coli* was obtained from the PubMLST database (http://pubmlst.org/campylobacter/). Sequenced *Campylobacter* genomes in either complete or draft form were downloaded from the GenBank database: *C. jejuni* subsp. *jejuni* NCTC 11168 (AL111168; NC_002163), *C. jejuni* subsp. *jejuni* 81116 (81116; NC_009839), *C. jejuni* subsp. *jejuni* 81-176 (81-176; NC_008787), *C. jejuni* RM1221 (RM1221; NC_003912), *C. jejuni* subsp. *jejuni* 84–25 (CJJ84-25; NZ_AANT00000000), *C. jejuni* subsp. *doylei* 269.97 (269.97; NC_009707), *C. jejuni* subsp. *jejuni* CF93-6 (CJJCF93-6; NZ_AANJ00000000), *C. jejuni* subsp. *jejuni* HB93-13 (CJJHB9313; NZ_AANQ00000000), *C. jejuni* subsp. *jejuni* CG8486 (CG8486; NZ_AASY00000000), *C. jejuni* subsp. *jejuni* CG8421 (CG8421; NZ_ABGQ00000000), *C. jejuni* subsp. *jejuni* 260.94 (260.94; NZ_AANK00000000) and *C. coli* RM2228 (AAFL00000000). In addition, the five *C. jejuni* plasmids pCJ419 (NC_004997), pCJ1170 (NC_008052), pCJ01 (NC_008438), pVir (NC_005012) and pTet (NC_007141) were also downloaded. The genomic characteristics of the sequences used in this study are described in more detail in Table S1, and the MLST profiles of these genomes are described in Table S2.

### Mapping of sequences

Short read sequences (in a modified Solexa FastQ format so that all fields were recorded in one row) were stored in a MySQL database for ease of retrieval. Prior to *de novo* assembly and mapping, the short reads were filtered using two steps. The first was to remove sequences that had any ambiguous bases (N) present. The second was to use the ‘fastx_artifacts_filter’ program from the FASTX toolkit (http://hannonlab.cshl.edu/fastx_toolkit/index.html) to remove artifactual sequences such as homopolymeric sequences. Initially the sequences were mapped with the proprietary mapper ELAND that exists as part of the Illumina pipeline. The filtered sequences were than mapped back to the reference genomes using Maq (version 0.7.1 [22]), and subsequently using BWA [23] for further mapping. In order to assess the quality of the base calling, and of the coverage of the short reads when mapped to the genome, the mapper BWA [23] was used allowing for a variety of mismatches (also known as an edit distances) in the mapping of the short reads to the reference genome AL111168. The resulting SAM files for the ST-474 isolates using an edit distance of 1 (the closest to default parameters with the short read sequence length used here) were parsed using the ‘pileup’ command within SAMtools [24] to generate a file allowing the nucleotide coverage at each base to be recorded. The process was repeated with edit distances of 0, 2, and 3. Parsing of this file allowed the calculation of the fraction of bases at a given nucleotide that were the same as the consensus base (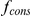). To visualise the data, heatmaps generated by the program matrix2png [25] were plotted showing how the value of 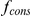 varied for the nucleotide coverage with a sliding window of 5 either side of a given nucleotide coverage.

### *de novo* assembly of sequences reads and draft genome assembly

The *de novo* assembler Velvet (version 0.7.55 [26]) was used for assembling the 36 base short reads. Due to the nature of the assembly process using de Bruijn graphs, the sequences from either P110b or H22082 were assembled across a range of kmers, for the odd numbers between 17 and 31 inclusive. These resulting Velvet contigs were then stored in the MySQL database. The minimus assembler (part of the AMOS package [27], [28]) was used in an attempt to reduce the contig numbers for the genomes of P110b and H22082. Each genome was dealt with separately. For this approach, the consensus sequence from the mapping of the short reads to the reference genome AL111168 was taken (effectively a scaffold), and broken up into contigs wherever any ambiguous bases were recorded in the consensus. These contigs were then assembled with the *de novo* contigs from a given kmer using minimus. In all, this meant that there were 8 new assemblies for each genome, all with fewer contigs. To check the validity of these new contigs, Maq was again used to map the short reads back to the contigs.

### Gene Prediction and Clustering

The concatenated minimus contigs generated at each kmer were run through the gene prediction program Glimmer (v3.02; [29]–[31]) to predict genes. All predicted genes were then analysed by both BLAST [32] and SSAHA2 [33] against 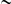
2.5 million bacterial genes in GenBank to see how many of the predictions matched known bacterial genes, especially those in the *Campylobacter* genus. Glimmer gene predictions were then clustered using OrthoMCL (version 1.4; [34], [35]) with the genes from the 11 reference strains.

### Sequence analysis and comparison of ST-474 genomes with each other and reference genomes

The core genome of the 11 reference strains plus the two new ST-474 genomes was calculated in order to evaluate the lineage of genes within ST-474. The clustering was performed using OrthoMCL [34], [35] on the predicted amino acid sequences of the genes in the 11 reference strains plus the predicted genes from the two ST-474 genomes for a total of 13 genomes. As defined here, a gene cluster had to have one, and only one member from each reference strain or ST-474 present, to be considered as a cluster. Cases where one strain was not represented occurred, but these were not considered further in this study. Each orthologous cluster was analysed in turn, so that the gene from one ST-474 isolate was aligned to each cluster member from the 11 reference strains using exonerate [36] with the ‘affine:local’ model to find the number of mismatches (i.e. sequence differences). A normalised sequence difference value for each of the 11 reference strains was calculated using the gene length (at either the DNA or protein level). The process was then repeated with the other ST-474 isolate for the same cluster. These values were then plotted against the respective ST-474 draft genome using the visualisation tool Circos [37].

The gene predictions from P110b and H22082 were clustered using OrthoMCL with the ‘pi_cutoff’ parameter using a value of 95%. A similar approach was taken as with the reference strains, only in these analyses, the gene from one ST-474 isolate was aligned to the orthologous gene of the other. The ‘affine:local’ model in exonerate was used to find both synonymous and non-synonymous sequence differences at both the DNA and protein level. These were again normalised for gene length. The genes that showed sequence differences between the strains were then localised in the genome, and small genomic regions with flanking genes were identified. In order to visualise these small regions with a consistent coordinate spacing, the genomic sequences (plus a flanking 50 bp) were extracted and a global alignment of the regions was performed using the program ‘stretcher’ (using a Needleman-Wunsch rapid global alignment algorithm) [38] in the EMBOSS package [39]. The genes from each ST-474 isolate were then mapped back to their adjusted genomic region using megablast, part of the BLAST package [32]. The genes from each ST-474 isolate with changed coordinates from these regions were then plotted with Circos in two ways; firstly to show their location of the regions in the genome, and secondly to show the normalised sequence difference rate for the genes.

In order to estimate the phylogenetic relationship between the *C. jejuni* reference genomes, and also of those to the two ST-474 strains, a large subsection of the *C. jejuni* core genome was used. For each gene cluster in the core genome, the length range of the orthologous cluster members was calculated. The sequences of the genes that showed the same length were used to generate a concatenated sequence for each of the 11 reference strains, and the two ST-474 strains. These sequences were generated at both the DNA and protein level. These sequences were then used to generate an unrooted phylogenetic network using the NeighborNet methodology within SplitsTree [40]. The parameters for NeighborNet visualisation were set using the NeighborNet distance transformation with the OrdinaryLeastSquares variance function, and the EqualAngle splits transformation using weights, and running a convex hull.

### Lineage and recombination analysis

A variant of the PACL approach [41], [42] was used to make inference about the ancestral lineage of the ST-474 isolates within each gene. This involves modelling the haplotype of a new isolate given the haplotypes of a sample of isolates. The model assumes that the new haplotype is a mosaic of the sample of isolates (due to recombination) together with nucleotide differences (due to mutation). The mosaic nature of the new haplotype describes the ancestral lineage of the corresponding isolate, and how that changes.

Briefly, under this model we calculate the conditional distribution of the ancestral lineages of one ST-474 isolate in terms of the other ST-474 isolate and the 11 reference genomes. We repeated this analysis for each gene and each of the ST-474 isolates. The output for a given gene and ST-474 isolate is a distribution on the ancestry of that ST-474 isolate, and how that ancestry changes (if at all) across the length of the gene. From this we can detect regions where recombination has imported new DNA since the common ancestor of the ST-474s, the position of these recombination events, and which nucleotide differences were introduced by recombination. As our method gives a distribution for these events, we can average over realisations from this distribution to calculate expected values (of say nucleotide differences introduced by recombination), or make probability statements (such as the probability of recombination within a given gene). We performed the analysis for each gene twice – once for each ST-474 isolate. Results given are averaged across these two analyses.

Assume we have a gene (or other fragment of the genome) consisting of 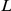 base-pairs. We have a sample of 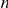 isolates for which we have the DNA sequence for this gene. That is, for each isolate we have the DNA base at each of the 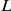 loci within the gene (we discuss how we deal with variations in sequence length below). Denote this haplotype information by a matrix 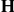, with 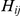 for 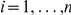 and 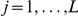 being the base at the 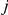
th position of the DNA sequence of the 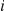
th isolate. We introduce a probability model for the DNA sequence of a new isolate. Denote the haplotype of this new isolate by 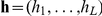, where 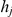 is the base at the 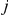
th position in the DNA sequence.

Our model is that the new haplotype is a mosaic of our sample of haplotypes, with additional mutations. We thus introduce a vector 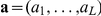 which denotes the ancestry of the new isolate (which of the other isolates the new isolate is most closely related to) at each position in the gene. We introduce a model for the ancestry, 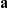, and for the conditional distribution of the haplotype of the new isolate, 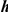, given the sample of haplotypes 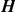 and its ancestry. We denote these by 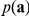 and 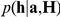 respectively. Once these have been defined we will aim to sample from the conditional distribution of 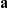 given 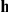 and 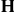 defined by
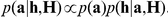
First we describe our model for 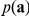. This model is based on the idea of an underlying clonal frame [2] in which the two ST-474 isolates will be most closely related. As in our application the new isolate is one of the ST-474 isolates, then it is most likely that at any position it would be most closely related to the other ST-474 isolate, and that these will be so closely related that the probability of any mutational difference will be small. However occasionally there will be recombination events at which fragments of DNA will have been imported into one of the two ST-474 isolates. These fragments will be mosaics of the haplotypes of the isolates in the sample 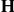. Within these fragments, the new isolate will be less closely related to its ancestral isolate, and thus there will a higher chance of mutation between it and its ancestral isolate.

Thus we allow 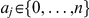, where 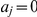 means that we are not within a recombinational fragment, and 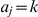 for 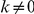 means that we are within a recombinational fragment and the ancestral isolate at this position is isolate 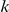. Note that we allow the ancestral isolate here to be the ST-474 isolate as well as the isolates of the reference genomes. We introduce a recombination probability per base-pair, 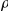, and a mean fragment length, 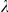, and recombination time 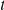, which describes the recombination process. Here 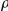 describes the probability of recombination when 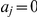, and 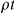 is the probability of recombination otherwise. Here 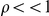 and 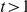 to model the fact that recombination is rare between the two ST-474 isolates, due to them being closely related, but that further recombination within a recombination fragment is more likely as the new ST-474 isolate is less closely related to its ancestral isolate within these fragments. Our model for the length of recombination fragment will be geometric. We further introduce a set of probabilities on isolates, 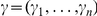, so that after a recombination event, the probability of the new ancestral isolate being isolate 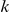 is 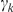.

Our model for 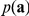 is then defined as a Markov model governed by the following transition probabilities
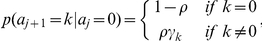
and for 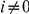,
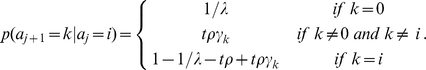
Finally 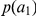 is defined to be the stationary distribution of the above Markov process.

Now we describe our model for 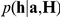. This is a simple mutational model whereby the new isolate is either an identical copy of the ancestral isolate at that position, or with small probability, is different due to a mutation. The mutation process is parameterised by 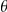 where 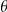 is the probability of mutation if 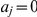 and 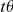 is the probability if 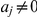.

Without loss of generality assume that the ST-474 isolate in 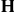 is the first isolate. We have that 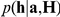 can be factorised as
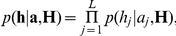
where
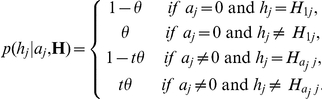
Our model is such that we can use the Forward-Backward algorithm [43] to sample from 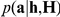.

For simplicity we ignored insertions and deletions within the data, by ignoring regions where there was an insertion or deletion within one or more of the DNA sequences within a gene or region. This avoids the need to separately model insertion/deletions and causes little loss of information as only 10% of sequence is removed.

To implement our procedure we need to fix the parameters of the model. Based on the mean divergence between the two ST-474 isolates (ignoring genes with 10 or more mutations difference, results in an average of 1 point mutational difference per 10 kb) we fixed 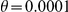. By comparison, the mean divergence between a ST-474 isolate and a reference genome is about 1%–2% so we fixed 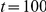. We estimate 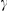 from the data using an EM algorithm. Based on previous analysis [7], [44] we set 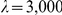 and 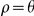. Whilst the latter choices are less clear, our results were robust to substantial variation about these.

To speed up computation we implemented a slight approximation of the above model which split genomic regions into sub-regions, with each sub-region containing one segregating site within the data set. We allowed the ancestry to change only at the boundaries of sub-regions. Transition of the ancestry for neighbouring sub-regions was calculated using the above model but taking into account the length of each sub-region. So for a sub-region of length 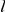 the transition probability for the ancestry of the next sub-region given the ancestry of the current one if 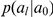, that is the 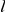-step transition probability of the above Markov process. This idea has been used previously [45]. The average length of sub-region was of the order of 10 bp, and this approximation introduced negligible difference.

The output of our analysis is drawn from 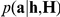. For a given realisation of 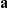 we can detect regions of recombination, where 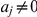, though this does not distinguish between which of the ST-474 isolates imported DNA via recombination. We can also detect recombination break-points, where either 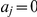 and 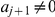 or 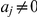 and 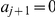. By averaging over realisations we can calculate probabilities of events (such as recombination within a gene), or averages (such as the number of recombination break-points).

### GenBank accession information

The P110b Whole Genome Shotgun project has been deposited at DDBJ/EMBL/GenBank under the accession AEIO00000000 (Genome Project ID 52015). The version described in this paper is the first version, AEIO01000000, with contigs labelled AEIO01000001 - AEIO01000029. The H22082 Whole Genome Shotgun project has been deposited at DDBJ/EMBL/GenBank under the accession AEIP00000000 (Genome Project ID 52017). The version described in this paper is the first version, AEIP01000000, with contigs AEIP01000001 - AEIP01000028. Illumina sequences have been deposited with the NCBI SRA database under the accession numbers SRA023887 and SRA023889 for P110b and H22082 respectively.

## Results

### Sequencing the genomes of the two ST-474 strains

An Illumina GAII was used to generate short read sequences of 36 bases for the two ST-474 strains using a lane for each isolate. 5389820 and 3272064 sequences were obtained for P110b and H22082 respectively, with a yield of 194.03 Mb and 117.79 Mb of sequence respectively. Using AL111168 as the closest complete reference genome for mapping [46], [47], and presuming that all short reads mapped back to a genome the size of AL111168, an average nucleotide coverage of 118.2 and 71.7 was found. The filtering procedures removed 74628 (1.38%) and 57424 (1.75%) sequences from P110b and H22082 respectively. Of these sequences, 38644 (0.72% of all sequences) and 19878 (0.61%) of these had ambiguous bases present, and 36009 (0.67%) and 37567 (1.15%) were sequences that were removed by the Fastx toolkit program ‘fastx_artifacts_filter’. The results of mapping the short read sequences to the 11 reference *C. jejuni* genomes and 5 plasmids from *C. jejuni* using the short read mapper Maq are shown in the Table S1.

### Mapping and gene prediction

There are three genomes to which the ST-474 short read sequences map best: AL111168, CJJCF93-6 and CJJ84-25, with an average value of 87.40% for P110b and 88.87% for H22082 (Table S1). It can also be seen that there is no appreciable mapping of the ST-474 reads to any of the known *C. jejuni* plasmids. It is known that within *C. jejuni* there is not 100% perfect mapping of short read sequences, due to the presence of three copies of the ribosomal RNA gene clusters, as well as paralogous genes such as *fla*A and *fla*B [48]. Even so, these differences in mapping frequencies for the ST-474 strains could not be accounted for, indicating that the ST-474 strains contained genomic sequences that were not found within the genome of AL111168.

In order to assess the nucleotide coverage of the short reads against the AL111168 genome, the mapper BWA [23] was used allowing for a variety of edit distances in the mapping. Figure S1 shows the results of these analyses of the BWA mappings using edit distances of 0, 1, 2 and 3 for H22082 (panels A and C) and P110b (panels B and D) against the AL111168 genome. In panels A and B the y-axis represents the number of bases, and the x-axis represents the nucleotide coverage for a given nucleotide position when mapped back to the reference genome AL111168. It can be seen that the overall coverage profiles of H22082 and P110b are very similar whether the mapping is performed with edit distances of 0, 1, 2, or 3 (black, red, green and blue lines respectively). There is, however, a slight shift to the right for the curves as more mismatches are allowed in the mapping. However, as more reads were generated for the P110b genome, the curves are moved to the right when compared to H22082. Using heatmaps, panels C and D show how the value for 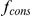 varies with the nucleotide base coverage. The nucleotide coverage range is the same as in panels A and B, and values are shown for edit distances of 1, 2 and 3 only, as by definition all bases will have 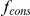 = 1.0000 with an edit distance of 0, and therefore are not plotted. Hence the short reads generated from both H220282 and P110b are of high quality.

The *de novo* ST-474 genomes are most similar to *C. jejuni* genomes with shared MLST alleles (as shown in Table S2). The de Bruijn graph assembler Velvet [26] was used to generate contigs from the short reads for the ST-474 isolates. A range of kmers were used for the assembly (Table S3). Using the genome assembler minimus and a combination of these contigs and a scaffold of sequences from the earlier mapping phase, the number of contigs was reduced dramatically (Table S3) without an appreciable change in overall calculated genome length. The contigs created with a kmer of 25 were chosen for further analysis after a consideration of a number of metrics to look at the *de novo* genomes (number of contigs generated by the minimus process, the percentage short reads hitting these contigs, the length of these contigs, maximal contig length and N50. In this process, the percentage of reads mapping to the new contigs using Maq increased to about 96% (data not shown).

The draft genomes of P110b and H22082 have 29 and 28 contigs, ranging in size from 147 bp to 369641 bp, and 494 bp to 310337 bp, with a total predicted length of 1.656341 Mb and 1.659123 Mb respectively. The N50s of the draft ST-474 genomes are 151393 bp and 138129 bp respectively. To get an overall view of the genomes of the ST-474 strains, they were visualised using the Artemis Comparison Tool (ACT) [49], [50] and AL111168 as the reference strain (Figure S2). There was a general degree of similarity in the overall architecture of the genomes, though even at this resolution, there were small regions of the ST-474 genomes that did not appear to map to AL111168. With this approach, it was also possible to see the overall similarity of the two ST-474 genomes to each other. It was also possible to visualise a novel gene insertion and/or deletion between the two ST-474 genomes, as explained in more detail below.

The MLST alleles were extracted for the 11 reference strains, and the resulting sequences were BLASTed against the PubMLST database for *Campylobacter spp.* The results of these analyses are shown in Table S2. ST-474 is part of clonal complex 48 (CC48) and whilst there is not a reference strain for this clonal complex, there are three strains for the related clonal complex CC21 (AL111168, CJJCF93-6 and CJJ84-25). These three strains share three, four and five MLST alleles with ST-474 respectively, and furthermore, these strains have the highest proportion of mapping short read sequences from the ST-474 genomes (Table S1).

The gene prediction algorithm Glimmer resulted in a different number of gene predictions for P110b (1687) and H22082 (1695). These numbers are well within the range seen for other *C. jejuni* genomes (Table S1). A significant portion (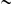
87%) of the gene predictions were identical in length to genes in *Campylobacter spp.*

To check for the presence of RNAs, the ST-474 contigs were tested for both the presence of rRNAs and tRNAs. Three copies of the rRNA cluster (5S, 23S and 16S rRNA) were found using the RNAmmer web server for ribosomal RNA prediction [51]. The tRNA-SE algorithm [52] was used for finding tRNAs. Both P110b and H22082 had 43 tRNAs for 20 amino acids, plus a tRNA for selenocysteine. Hence, the finding of 53 RNAs (9 rRNAs and 44 tRNAs) in both ST-474 genomes is comparable with what is seen with the 11 reference strains: for example, there are 56 RNAs reported for AL111168. It was thus concluded that the ST-474 draft genomes were relatively complete, and therefore the detailed gene lineage analysis could be undertaken without completion of full genome assemblies.

### The ‘core’ genome

**Figure 1:**
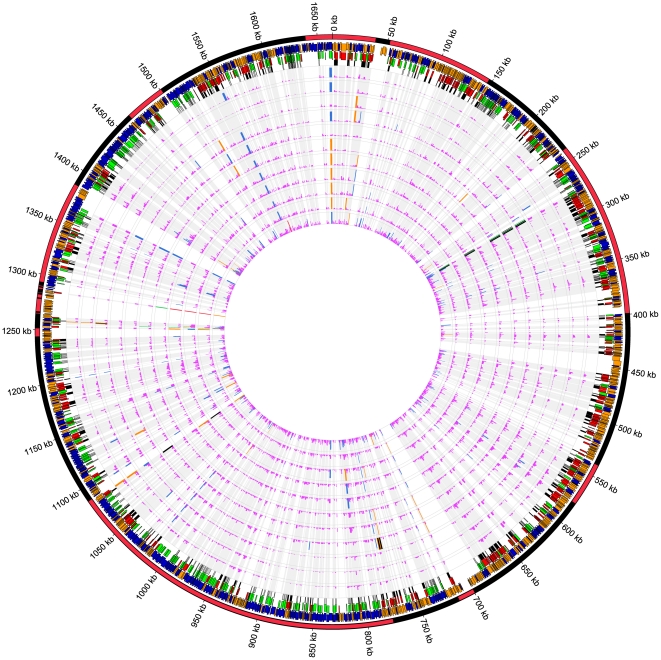
Circos plot showing the sequence differences detectable at the protein level for *C. jejuni* strain H22082. The figure shows the location of the core genome genes as located within the H22082 draft genome when compared to the core genomes of 11 *C. jejuni* reference strains. The tracks from outside to inside are; chromosomal ideogram (alternating colours indicate contigs), Glimmer gene prediction on the whole genome (forward genes in orange, and reverse genes in blue); core genome genes (forward genes in red, and reverse genes in green); core genome genes that are the same length (forward genes in black, and reverse genes in grey); 11 histograms showing the number of sequence differences detectable as a fraction of protein length against the genomes in the following order: CJJ84-25, AL111168, CJJCF93-6, CG8421, CG8486, RM1221, CJJHB9313, 81-176, 81116, 260.94 and 269.97. For clarity, the histograms have been scaled to cover the range 0 to 0.10 (purple 0 to 0.05 and blue 0.05 to 0.10). The values above 0.10 are plotted as a full scale value with colours indicating the scale: orange (0.10 to 0.20), black (0.20 to 0.30), green (0.30 to 0.40) and red (above 0.40).

We estimated the core genome of the isolates under investigation using OrthoMCL version 1.4 [34]. This program has a number of parameters, and we investigated the effect of these on the inference of the core genome. Only the ‘pmatch’ (equivalent to sequence similarity) and the ‘pi_cutoff’ (similar to sequence identity) parameters affected gene clustering, with the former having more of an effect than the latter. This was because the clustering was performed on orthologous genes from strains of the same species. As we varied ‘pi_cutoff’ from 80% to 100% the number of genes decreased from 1069 to 25. The ‘pmatch’ parameter was chosen for further investigation as this allowed for sequence changes to occur, but resulted in protein sequences that could have been identical. The numbers of genes changed from 1071 for a ‘pmatch’ parameter value of 80% to 836 for a value of 100%. We chose a value of 95% which resulted in 1001 clusters in the core genome. Using the visualisation tool Circos, these 1001 genes are shown graphically in Figure 1 with reference to the draft genome of H22082 at the protein level. The number of differences per gene normalised for gene length is shown as histograms for each pairwise comparison. This figure is also provided as a high resolution large image (Figure S3). Similar analyses of P110b at the DNA level, and H22082 at both the DNA and protein level are shown in Figures S4, S5 and S6 as large high resolution images. The 1001 genes identified here as the ‘core genome’ accounted for between 54.5% and 70.3% of genes in the genomes under investigation. For the reference genome AL111168, 500 genes were identified on the positive strand and 501 on the negative strand. Genes in the core genome of AL111168 are distributed relatively evenly across the genome, with a few notable exceptions, as shown in Figure 1.

**Figure 2:**
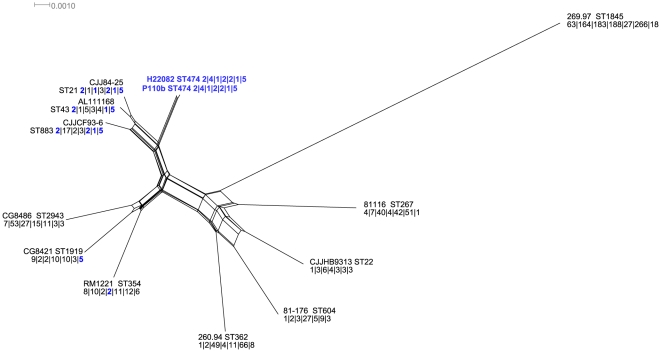
The unrooted NeighborNet network of the two ST-474 strains and the 11 reference*C. jejuni* strains. The two ST-474 strains (P110b and H22082) are shown in bold blue text. The network is at the DNA level (430641 bases), using 477 of the 1001 genes that have orthologous gene members of the same length. For each genome, the name as well as the sequence type (ST) and allele profile is shown. The alleles the reference genomes share with those in ST-474 are indicated in blue bold text.

In order to generate an order for the relatedness of the core genomes, 477 genes were analysed where there was no difference in predicted gene length. Sequence differences were then calculated between each of the two ST-474 isolates and the reference strains to give an order that was subsequently used for further analyses (Table S4), for example in the core genome visualisation. As would be expected, there is also a strong correlation between this order and a phylogenetic network that was generated from a concatenated sequence of these 477 genes. A NeighborNet tree showing the phylogenetic relationships between the two ST-474 and the 11 reference genomes in Table S4 is shown in Figure 2.

When the gene predictions from just the two ST-474 genomes were clustered, there were 1568 genes that formed orthologous gene pairs, highlighting how sensitive orthologous gene clustering methods are to the relatedness of the input gene sequences. For example, when OrthoMCL clustering is performed with an additional five non *C. jejuni* genomes (*C. coli*, GI 57505198; *C. consicus*, GI 157163852; *C. curvus*, GI 154173617; *C. fetus*, GI 118474057 and *C. hominis*, GI 154147866) to generate a *Campylobacter spp.* core genome set, the number of genes, and hence the size of the *Campylobacter spp.* core genome drops, to 482 at a ‘pmatch’ value of 95% (data not shown).

### Lineage analysis of gene predictions

**Figure 3:**
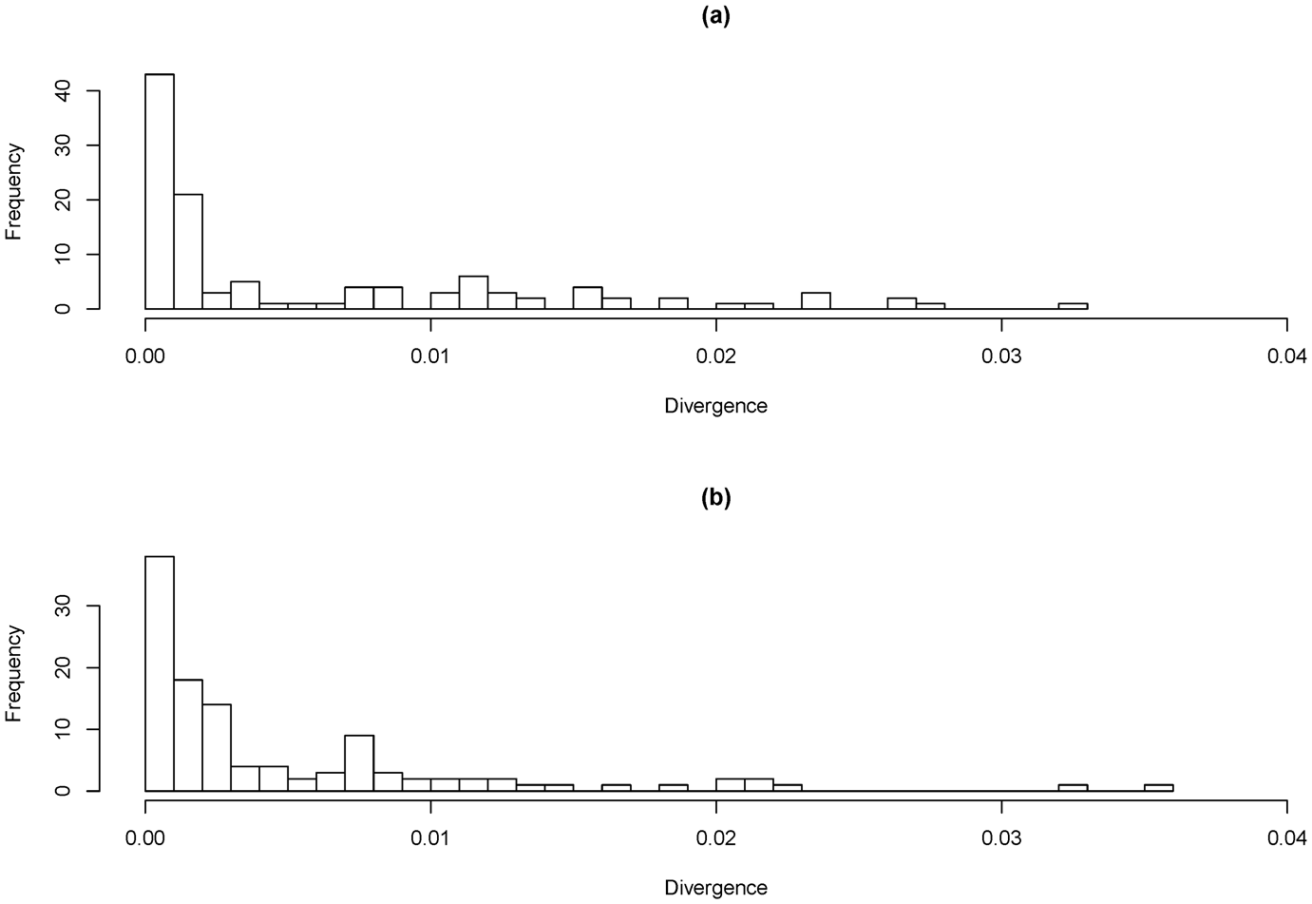
Plot of frequency of mutational differences per gene. (a) Differences are shown for the two ST-474 isolates and, (b) of H22082 against the reference genome. Divergence shown for the genes at which the two ST-474 differed. For the latter comparison, each gene was compared with the reference genome whose gene sequence was the most similar.

We analysed data from 1502 genes for which we had sequences within both ST-474 isolates and within at least one reference genome. The average gene length was 920 bp, ranging from 111 bp to 4488 bp. For 94.5% of the genes, the two ST-474s isolates were identical. The divergence between the two ST-474s for the remaining genes is shown in Figure 3(a). We also compared the ST-474s with the sequences from the reference genomes. For each gene we compared with the reference genome whose sequence was most similar. For 49% of gene comparisons, the ST-474 was identical to the closest reference genome. For the genes where the two ST-474s differed, the divergence of H22082 with the closest reference genome is shown in Figure 3(b).

From Figure 3 we see a large tail for the number of differences between the two ST-474 isolates. We investigated whether this was due to recombination, by estimating how the ancestral lineage of each ST-474 varies across each gene. This method enables us to calculate the probability that a given region of a gene has undergone recombination in at least one of the ST-474s since their common ancestor. An example of the output obtained from the algorithm is shown in Figure S7. For such recombinant regions it gives a distribution on which of the reference genomes was the source of the sequence imported – though it cannot infer which of the ST-474s imported DNA due to recombination. It also cannot distinguish between multiple overlapping recombination events, and a single recombination event which imported sequence that is a mosaic of the reference sequences.

**Figure 4:**
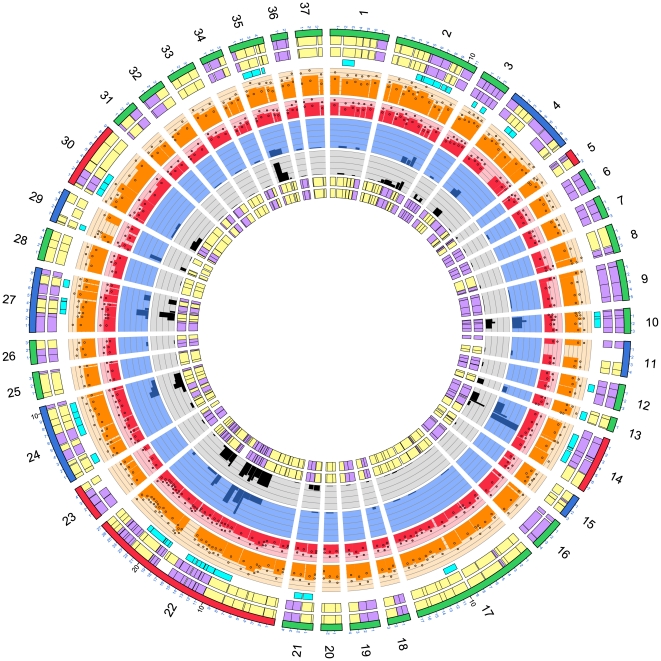
Circos plot of orthologous genomic regions for the two ST-474 genomes where sequence differences are found. The figure shows 37 detectable regions. Orthologous gene pairs in P110b and H22082 were calculated using OrthoMCL with a ‘pi_cutoff’ value of 0.95. The tracks from outside to inside are; genome region name and size (green, regions of same length in P110b and H22082; blue, the region from P110b is longer; red, the region from H22082 is longer); genes and sizes for P110b; genes and sizes for H22082; genes coloured cyan showing evidence of recombination; histograms for P110b (orange) and H22082 (red) showing the nucleotide coverage from the short reads plotted as an average over the length of the gene, along with the standard deviations for the coverage as round circles; histograms showing the number of sequence differences between the genes at the protein level (dark blue on a light blue background) and DNA level (black on a grey background) as a fraction of protein or gene length respectively; a repeat of the gene sizes and locations in P110b and H22082. Gene orientations are shown in purple and yellow for the forward and reverse strand respectively. The nucleotide coverage histograms have the same scale but a different magnitude, 180 and 120 for P110b and H22082 respectively. Similarly the sequence difference histograms have the same scale but a different magnitude, 0.05 and 0.04 for the protein and the DNA histograms respectively.

We analysed 83 genes where there were point differences between the two ST-474 sequences. Their location relative to the P110b draft genome is shown in Figure S8. The 83 genes are located within 37 regions, and these are plotted, along with a flanking gene for clarity, in Figure 4. These regions are distributed relatively evenly throughout the genome. Twenty four of these regions have a single gene, and the remaining genes are found in 13 groups of varying genomic size, involving two to 14 genes showing sequence differences. Fourteen regions have at least two neighbouring genes showing sequence differences, and the remainder are genes that group together in localised genomic regions. The genes that show differences between the strains cover a broad range of cellular functions, and do not appear to be enriched for any particular group.

These genes covered 101 kb and contained 672 point differences. Further information regarding these 672 point differences is shown in Table S5, including the nucleotide coverage at the affected base in the orthologous genes from P110b and H22082. The orthologous gene in a reference genome, which is AL111168 for all but two genes where it is CJJ84-25, is also shown. The mean and median nucleotide coverage values are 104.8 and 101 for H22082, and 68.2 and 66 for P110b respectively. Similarly, the mean and median 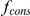 values are 0.9944 and 1.0000 for H22082, and 0.9947 and 1.0000 for P110b respectively. These data indicate that the bases where differences between H22082 and P110b are observed have been sequenced to a sufficient coverage, and that the presence of a small number of random sequencing errors (a known artefact with Illumina sequencing) is not affecting the results. Hence for a given nucleotide location where there are differences between the ST-474 isolates the value of 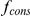 is very high (over 0.85). There are two exceptions to this, in the gene *Cj1136* for P110b and the gene *Cj1340c* in H22082 where 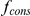 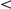
0.5. *Cj1136* is part of the lipooligosaccharide locus, and *Cj1340c* is part of the O-linked glycosylation locus, which are both sources of known hypervariability within the *C. jejuni* genome.

Of these 672 point differences, we inferred that 22 (3.3%) were due to mutation, and 650 (96.7%) were imported via recombination. This suggests that recombination has had a much larger effect on the diversity of the ST-474 isolates, which is different from results in Vos and Didelot [53] who estimated that in *C. jejuni* recombination introduced only about twice as many point differences as mutation. In total we estimated recombination imported 44 kb of sequence within these genes. We found strong evidence of recombination (probability of recombination 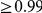) within 51 genes, with these genes located within 16 of the 37 regions. We found evidence for both large regions affected by recombination, with instances of 11 neighbouring genes and 7 neighbouring genes showing strong evidence of recombination; as well as 4 cases of recombination affecting only single genes.

Of particular interest was the fact that in the H22082 strain, two extra genes were inserted in one of the large regions with evidence for recombination. Analysis showed that these two genes were only found in one reference strain, that of CJJ84-25. These genes were not present elsewhere in the genome of P110b, indicating that these genes had been inserted into the H22082 genome through a recombination event, or had been lost from P110b by a similar mechanism. The two inserted genes show high similarity to *ykg*C, and a gene which is defined as “domain of unknown function superfamily *DUF302*” in the Pfam database [54].

Our results give further partial information about the recombination process itself. Our analysis inferred an average of 38 recombinant breakpoints, which would correspond to at least 19 recombination events. This gives a lower-bound on the number of recombination events, as it ignores recombination breakpoints which occurred outside the 83 genes we analysed; and also would not detect all breakpoints if we have had overlapping recombination events. However this suggests a recombination rate of a similar order of magnitude to the mutation rate (consistent with results in Fearnhead et al. [44]). It also gives a very rough estimate of average tract length of 2.3 kb – with 44 kb of imports due to the 19 events. This is larger than estimated in Fearnhead et al. [44], but similar to results in Schouls et al. [55].

We also looked at the inferred ancestry of the recombinant fragments. The proportion of the recombinant fragments that can be allocated to each of the reference genomes is shown in Table 1. Note that we cannot distinguish between the ancestry of recombinant imports, and the ancestry of the sequence replaced by these imports (which would be observed on the ST-474 isolate that did not undergo recombination). However, from Figure 2, we note that in recombinant regions, the sequence that is not imported would be expected to be most similar to one of the CJJ84-25, CJJCF93-6 or AL111168 sequences. Within these regions, we inferred that the poultry isolate (P110b) ancestry was from one of these three isolates 51% of the time, whereas the human isolate (H22082) only 29% of the time. While it is difficult to draw strong conclusions, this suggests that recombination has imported more DNA into H22082 than into P110b.

**Table 1:** Proportion of ancestry (as %) of recombinant fragments for each ST-474 that can be associated with each reference genome.

Locus ID	Genome Project	Genome Name	H22082	P110b
NZ_AANK00000000	subsp. jejuni 260.94	260.94	6	6
NZ_ABGQ00000000	subsp. jejuni CG8421	CG8421	7	9
NZ_AASY00000000	subsp. jejuni CG8486	CG8486	4	9
NZ_AANT00000000	subsp. jejuni 84-25	CJJ84-25	12	16
NZ_AANJ00000000	subsp. jejuni CF93-6	CJJCF93-6	6	18
NZ_AANQ00000000	subsp. jejuni HB93-13	CJJHB9313	15	5
NC_002163	subsp. jejuni NCTC 11168	AL111168	11	17
NC_003912	RM1221	RM1221	11	9
NC_008787	subsp. jejuni 81-176	81-176	18	5
NC_009839	subsp. jejuni 81116	81116	8	6
NC_009707	subsp. doylei 269.97	269.97	2	0

## Discussion

We have used the draft genome from each of two closely related rare MLST *C. jejuni* isolates to investigate lineage differences between these strains. Using a model based on the PACL approach [41], [42], we have been able to make inferences about the ancestral lineage of the ST-474 isolates within each gene where the orthologue in a reference strain can be determined. We have found that genomes that appear similar at the MLST level (i.e. those sharing MLST alleles with ST-474 at the seven housekeeping genes used), or at a core genome level (i.e. those closest to ST-474 in the NeighborNet analysis) are those that are more likely to be the source of the recombined genes in ST-474.

Our two samples have been typed by MLST and *fla*A SVR typing [10], [11] as being ST-474 *fla*A SVR-14. This makes these genomes identical by these typing methods. However, the introduction of high throughput sequencing methodologies means that it is possible to analyse the genomes of bacteria in great detail, and we have shown that a small percentage of the genes in the genome (55 genes; 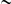
3.2%) show differences between their genomes that result in non-synonymous substitutions. The functional implications of these non-synonymous substitutions have yet to be elucidated.

We have found 37 regions of the ST-474 genome where sequence differences are observed between H22082 and P110b. Of these regions, the largest region showing evidence of recombination showed two extra genes in H22082 that were not found in P110b. Whilst one of these two genes has minimal annotation associated with it, the other gene – *ykg*C, a putative oxidoreductase – has been studied. In a study examining the induction of *E. coli* gene promoter elements on exposure to seawater, genes under control of RpoS predominated the set [56]. RpoS is the 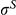 subunit of RNA polymerase governing expression in response to stress and one of the responsive genes was *ykg*C. Therefore, it is possible that the acquisition or loss of *ykg*C in ST-474 provides a selective advantage to the isolate enabling it to respond to a new or fluctuating environment, such as transmission to a new host. Recently it has been shown that widespread genome changes can be found in *C. jejuni* genomes from a variety of environmental sources, and furthermore, that such divergence may provide evidence of adaptation leading to niche specialisation [57]. It has also been shown that there is a relationship between ecological factors and population structure. In comparing the MLST alleles found in wild birds to those in farmed poultry, it has been shown that the wild birds carry phylogenetically distinct alleles to those found in poultry flocks [58]. There was also a greater admixture of alleles found amongst all farm animals, suggesting that there may be a ‘farm-type’ *C. jejuni* within this agricultural niche, and selection for such genotypes transcends the host species [58].

The samples used in this study were part of a public health investigation because of New Zealand's high campylobacteriosis rates, mostly due to poultry, according to source attribution studies. It is now becoming clear that the landscape of campylobacteriosis in New Zealand is changing due to interventions put in place in the poultry industry, and that the rates are dropping because of these interventions [59], [60]. However despite this success, campylobacteriosis is still a problem in New Zealand, with levels higher than most industrialised countries [60], although the epidemiology is showing evidence of changing. Recently it has been shown that common *Campylobacter spp.* genotypes found commercially are also present in ‘backyard’ chicken flocks to a high prevalence [61], showing that there might be other transmission pathways for human infection.

In the analyses described here, we have inferred that the main driver of diversity for the ST-474 isolates is recombination. Firstly, we have observed the introduction of new genes by recombination. Furthermore, while we infer the number of regions where new DNA has been imported by recombination is similar to the number of point mutations, on average these regions are of the order of a few kilobases in length and introduce of the order of 10 nucleotide differences. Overall we infer that 97% of single nucleotide changes in the DNA have been introduced by recombination as opposed to mutation.

This estimate is markedly higher than that of Vos and Didelot [53], who estimate that recombination introduces approximately twice as many nucleotide changes than does mutation. There are a number of possible explanations for this difference. Firstly, we have looked only at the differences between two isolates. The overall number of mutation and recombination events is still of the order 50. So the very large difference – we would require nearly 10 times as many mutation events or 1/10th as much recombination to account for the difference with Vos and Didelot [53] – suggests that this is unlikely by chance unless single recombination events can introduce new DNA at multiple regions.

Secondly, Vos and Didelot [53] analysed data just in the MLST housekeeping genes whereas we have looked at the whole genome. It may be that the patterns of the MLST housekeeping genes are different from other genes, for example with lower recombination rates, or recombination events introducing fewer nucleotide differences. Finally, as we have looked at divergence between two closely related isolates, there has been less opportunity for purifying selection to have removed weakly deleterious changes. It is likely that the larger changes introduced by recombination are more likely to be deleterious, and thus the relative effect of recombination on differences between less closely related isolates (such as those studied by Vos and Didelot [53]) would be smaller. One approach to testing between these latter two possibilities, would be to reanalyse MLST data but to focus on differences between closely related isolates, such as pairs of isolates that differ at a single MLST locus.

There is a substantial literature based on the analysis of MLST alleles in *C. jejuni* and *C. coli*, due to their genomic conservation, which is estimated to be 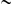
85% and as a direct consequence, their common MLST scheme. Many important findings on evolution within *Campylobacter spp.* have been derived from these analyses. For example, it has been hypothesised that these species are converging, most likely due to agricultural intensification [6], although three distinct *C. coli* clades were identified in this study. This is not a reciprocal relationship, with *C. coli* importing DNA from *C. jejuni* [6], [62]. One of these three clades – clade 1 – has been shown to be responsible for the genotypes found in farm animals and those that cause disease in humans [63]. Due to the relatedness of *C. jejuni* and *C. coli*, mosaic alleles, that is alleles having portions of their sequence having different origins, can be detected. These can act as a marker to show the spread and movement of genetic material between these two species. Combining these two concepts, of introgression and mosaic alleles, a large scale study of MLST sequences has recently shown that it is *C. coli* clade 1 that has acquired alleles from *C. jejuni*, and the other two *C. coli* clades have not [62]. Despite the power of the MLST approach in analysing many isolates at a few loci, the cost disparity between preparing MLST alleles for capillary sequencing and routinely sequencing whole bacterial genomes is continuously reducing. Hence information about the complete sequence of the seven genes used for MLST, in addition to the currently used PCR products for the central portion can be analysed, as well as many other genes, as described here.

To perform the lineage analysis a core genome of 1001 genes was generated for the *C. jejuni* ST-474 isolates and 11 reference strains found in GenBank at the time of the analysis. The core genome of the species has been estimated by looking at genomes of already sequenced *C. jejuni* strains. A recent value was 1295 [21]. The parameters chosen by Friis et al. to include genes in the core genome were not as conservative as those reported here (a significant hit had an alignment covering at least 50% of both sequences with at least 50% identity), so it is not surprising that our estimation of the *C. jejuni* core genome is smaller. Another recent paper has sequenced 42 *C. jejuni* strains and found a core genome size for this species of 1325 core genes [64]. Lefebure et al. also applied a slightly more liberal approach to that described here by allowing their core genes to be missing in one of the strains under analysis. If this criterion was applied to our dataset, the core genome size would increase by 252 core genes to 1253.

A different approach to core genome estimation has been to use mathematical models to estimate the size of the core genome if it were possible to sequence an infinite number of isolates for a given species [20]. Using this approach, the *C. jejuni* core genome is 847 genes, indicating there are many more genes to be discovered for *C. jejuni*. The parameters for defining gene families used by Friis et al. [21] are the same as those used in this paper. Many of the new genes would be character and accessory genes, thereby reducing the size of the core genome as they are discovered in the future. Considering *Campylobacter spp.*, the core genome has been estimated to comprise 647 genes [18]. This compares more favourably to our estimation of the *Campylobacter spp.* core genome being 482 with our more conservative parameters.

To conclude, this study has provided new insight into the evolution of *C. jejuni*, and the processes that have generated sequence variation in multilocus sequence type ST-474; the most prominent enteric pathogen in New Zealand. In analysing two *C. jejuni* isolates that are genotyped to be identical using currently typing schemes, we provide evidence for multiple lateral gene transfer events, importing tracts of DNA from diverse lineages of *C. jejuni* into ST-474 via both homologous and non-homologous recombination. The ability to uptake and insert naked DNA into the *C. jejuni* genome may be an important mechanism for population-level adaptation to fluctuating environments, such as those encountered during transmission between different host species.

## Supporting Information

Figure S1
**Nucleotide coverage plots showing nucleotide coverage and consensus base summary statistics from BWA mappings for the** ***C. jejuni*** **strains P110b and H22082.** In the figure the results of mapping the short reads with BWA to the draft contigs are shown using an edit distance of 0 to 3 in the mapping (0: black, 1: red; 2: green; and 3: blue). Panels A and B show the number of bases in the genome at each nucleotide coverage for H22082 and P110b respectively. The average nucleotide coverages are 54.6, 64.1, 66.5 and 67.2 for H22082, and 89.5, 102.4, 106.5 and 107.8 for P110b for edit distances of 0, 1, 2 and 3 respectively. 99.56%, 99.81%, 99.85% and 99.86% of the genomic bases for H22082 have a nucleotide coverage of 20 or more for mapping with edit distances of 0, 1, 2 and 3 mismatches. The analogous numbers for P110b are 99.97%, 99.99%, 99.99% and 99.99%. Panels C and D show three heatmaps to show how 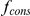 varies for edit distances of 1, 2 and 3 for the nucleotide coverage. Colours go from white (0.0000) through a black body radiation heatmap to black (1.0000). Each row represents the values for a sliding window of 5 either side of each nucleotide coverage value. All nucleotide coverage values from 10 to 200 are plotted for H22082, and the values for 10 to 250 for P110b. The six columns from left to right labelled as ‘I’, ‘ii’, ‘iii’, ‘iv’, ‘v’ and ‘vi’ represent 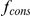 values in the following groups, and for the following ranges: 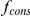 = 1.0000; 0.9800 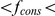 0.9999; 0.9500 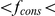 0.9799; 0.9000 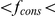 0.9499; 0.8000 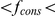 0.8999; 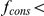 0.8000. In this way, all rows add up to 1.0000.(TIF)

Figure S2 **ACT plots showing how the two ST-474 genomes relate to the reference genome AL111168.** Genomes from top to bottom are P110b, AL111168 and H22082.
(TIF)

Figure S3 **Circos plot showing the sequence differences detectable at the protein level for** ***C. jejuni*** **strain H22082.** The figure shows the location of the core genome genes as located within the H22082 draft genome when compared to the core genomes of 11 *C. jejuni* reference strains. The tracks from outside to inside are; chromosomal ideogram (alternating colours indicate contigs), Glimmer gene prediction on the whole genome (forward genes in orange, and reverse genes in blue); core genome genes (forward genes in red, and reverse genes in green); core genome genes that are the same length (forward genes in black, and reverse genes in grey); 11 histograms showing the number of sequence differences detectable as a fraction of protein length against the genomes in the following order: CJJ84-25, AL111168, CJJCF93-6, CG8421, CG8486, RM1221, CJJHB9313, 81-176, 81116, 260.94 and 269.97. For clarity, the histograms have been scaled to cover the range 0 to 0.10 (purple 0 to 0.05 and blue 0.05 to 0.10). The values above 0.10 are plotted as a full scale value with colours indicating the scale: orange (0.10 to 0.20), black (0.20 to 0.30), green (0.30 to 0.40) and red (above 0.40).
(EPS)

Figure S4 **Circos plot showing the sequence differences detectable at the DNA level for** ***C. jejuni*** **strain H22082.** The figure shows the location of the core genome genes as located within the H22082 draft genome when compared to the core genomes of 11 *C. jejuni* reference strains. The tracks from outside to inside are; chromosomal ideogram (alternating colours indicate contigs), Glimmer gene prediction on the whole genome (forward genes in orange, and reverse genes in blue); core genome genes (forward genes in red, and reverse genes in green); core genome genes that are the same length (forward genes in black, and reverse genes in grey); 11 histograms showing the number of sequence differences detectable as a fraction of protein length against the genomes in the following order: CJJ84-25, AL111168, CJJCF93-6, CG8421, CG8486, RM1221, CJJHB9313, 81-176, 81116, 260.94 and 269.97. For clarity, the histograms have been scaled to cover the range 0 to 0.10 (purple 0 to 0.05 and blue 0.05 to 0.10). The values above 0.10 are plotted as a full scale value with colours indicating the scale: orange (0.10 to 0.20), black (0.20 to 0.30), green (0.30 to 0.40) and red (above 0.40).
(EPS)

Figure S5 **Circos plot showing the sequence differences detectable at the protein level for** ***C. jejuni*** **strain P110b.** The figure shows the location of the core genome genes as located within the H22082 draft genome when compared to the core genomes of 11 *C. jejuni* reference strains. The tracks from outside to inside are; chromosomal ideogram (alternating colours indicate contigs), Glimmer gene prediction on the whole genome (forward genes in orange, and reverse genes in blue); core genome genes (forward genes in red, and reverse genes in green); core genome genes that are the same length (forward genes in black, and reverse genes in grey); 11 histograms showing the number of sequence differences detectable as a fraction of protein length against the genomes in the following order: CJJ84-25, AL111168, CJJCF93-6, CG8421, CG8486, RM1221, CJJHB9313, 81-176, 81116, 260.94 and 269.97. For clarity, the histograms have been scaled to cover the range 0 to 0.10 (purple 0 to 0.05 and blue 0.05 to 0.10). The values above 0.10 are plotted as a full scale value with colours indicating the scale: orange (0.10 to 0.20), black (0.20 to 0.30), green (0.30 to 0.40) and red (above 0.40).
(EPS)

Figure S6 **Circos plot showing the sequence differences detectable at the DNA level for** ***C. jejuni*** **strain P110b.** The figure shows the location of the core genome genes as located within the H22082 draft genome when compared to the core genomes of 11 *C. jejuni* reference strains. The tracks from outside to inside are; chromosomal ideogram (alternating colours indicate contigs), Glimmer gene prediction on the whole genome (forward genes in orange, and reverse genes in blue); core genome genes (forward genes in red, and reverse genes in green); core genome genes that are the same length (forward genes in black, and reverse genes in grey); 11 histograms showing the number of sequence differences detectable as a fraction of protein length against the genomes in the following order: CJJ84-25, AL111168, CJJCF93-6, CG8421, CG8486, RM1221, CJJHB9313, 81-176, 81116, 260.94 and 269.97. For clarity, the histograms have been scaled to cover the range 0 to 0.10 (purple 0 to 0.05 and blue 0.05 to 0.10). The values above 0.10 are plotted as a full scale value with colours indicating the scale: orange (0.10 to 0.20), black (0.20 to 0.30), green (0.30 to 0.40) and red (above 0.40).
(EPS)

Figure S7 **Output for our algorithm for one gene with strong evidence for recombination.** The top plot shows the inferred ancestry of P110b, the bottom for H22082. Each row in each plot corresponds to a state of our ancestral process. Below the bold dashed line corresponds to a non-recombinant fragment; above the bold dashed line corresponds to recombinant fragment with the ancestral lineage being the corresponding isolate. Stars denote mutational differences of each of the haplotypes with the new ST-474 isolate. The coloured lines correspond to 5 realisations of the ancestral process.
(EPS)

Figure S8 **Circos plot showing the detectable regions where there are sequence differences between P110b and H22082.** The P110b draft genome is shown indicating the genomic location of the 37 gene regions centred around one or more genes where sequence differences are detectable. The tracks from outside to inside are; chromosomal ideogram (alternating colours indicate draft contigs), region location with number (as in Figure 4) and Glimmer gene predictions on the whole genome (forward genes in orange, and reverse genes in blue), and core genome genes (forward genes in red, and reverse genes in green).
(EPS)

Table S1 **Genome characteristics of 11 reference genomes and 5 plasmids from** ***C. jejuni.***
(XLSX)

Table S2 **MLST profiles for the** ***C. jejuni*** **strains.**
(XLSX)

Table S3 ***de novo*** **assembly metrics for P110b and H22082 using Velvet and minimus.**
(XLSX)

Table S4 **The number of sequence differences between the two ST-474 isolates and each of the eleven** ***C. jejuni*** **reference strains.**
(XLSX)

Table S5 **Details of the 672 SNP differences found within 83 genes between the ST474 strains P110b and H22082.**
(XLSX)
