# Supplementary figures and images for: Whole-Genome Comparison of Two Campylobacter jejuni Isolates of the Same Sequence Type Reveals Multiple Loci of Different Ancestral Lineage
Source: PLoS One. 2011 Nov 11;6(11):e27121. doi: 10.1371/journal.pone.0027121 (PMC3214069; doi:10.1371/journal.pone.0027121)

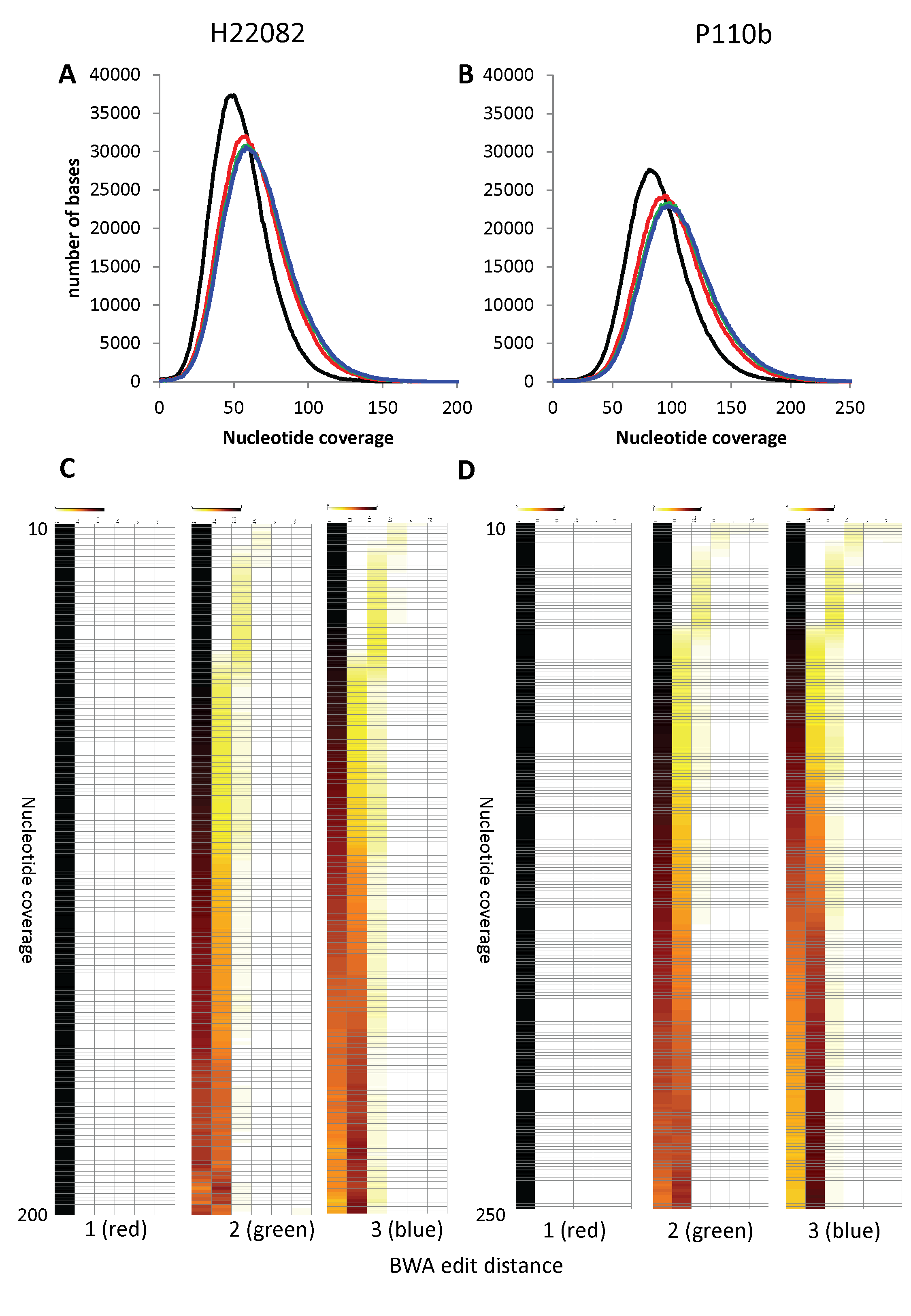

Supplement: Figure S1 — Nucleotide coverage plots showing nucleotide coverage and consensus base summary statistics from BWA mappings for the C. jejuni strains P110b and H22082. In the figure the results of mapping the short reads with BWA to the draft contigs are shown using an edit distance of 0 to 3 in the mapping (0: black, 1: red; 2: green; and 3: blue). Panels A and B show the number of bases in the genome at each nucleotide coverage for H22082 and P110b respectively. The average nucleotide coverages are 54.6, 64.1, 66.5 and 67.2 for H22082, and 89.5, 102.4, 106.5 and 107.8 for P110b for edit distances of 0, 1, 2 and 3 respectively. 99.56%, 99.81%, 99.85% and 99.86% of the genomic bases for H22082 have a nucleotide coverage of 20 or more for mapping with edit distances of 0, 1, 2 and 3 mismatches. The analogous numbers for P110b are 99.97%, 99.99%, 99.99% and 99.99%. Panels C and D show three heatmaps to show how varies for edit distances of 1, 2 and 3 for the nucleotide coverage. Colours go from white (0.0000) through a black body radiation heatmap to black (1.0000). Each row represents the values for a sliding window of 5 either side of each nucleotide coverage value. All nucleotide coverage values from 10 to 200 are plotted for H22082, and the values for 10 to 250 for P110b. The six columns from left to right labelled as ‘I’, ‘ii’, ‘iii’, ‘iv’, ‘v’ and ‘vi’ represent values in the following groups, and for the following ranges: = 1.0000; 0.9800 0.9999; 0.9500 0.9799; 0.9000 0.9499; 0.8000 0.8999; 0.8000. In this way, all rows add up to 1.0000. (TIF) [file pone.0027121.s001.tif]

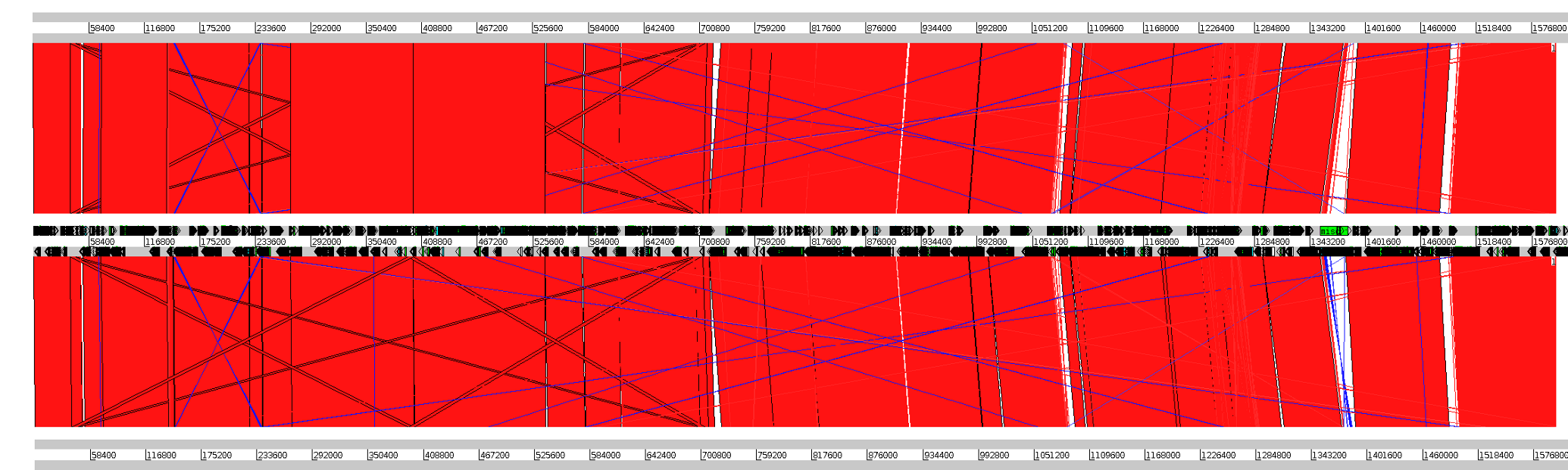

Supplement: Figure S2 — ACT plots showing how the two ST-474 genomes relate to the reference genome AL111168. Genomes from top to bottom are P110b, AL111168 and H22082. (TIF) [file pone.0027121.s002.tif]
